# (*E*)-1-[(2-Chloro-5-methyl­pyridin-3-yl)methyl­ene]thiosemicarbazide

**DOI:** 10.1107/S1600536810004915

**Published:** 2010-02-13

**Authors:** Zhen Wang, Yongqiang Ma, Yan Xu, Yun Ling, Xinling Yang

**Affiliations:** a College of Science, China Agricultural University, Beijing, 100193, People’s Republic of China.

## Abstract

The title compound, C_8_H_9_ClN_4_S, which has potential insecticidal activity, was synthesized by the reaction of 2-chloro-5-methyl­nicotinaldehyde and thio­semicarbazide. In the crystal structure, the mol­ecules are linked *via* inter­molecular N—H⋯N, N—H⋯S and N—H⋯Cl hydrogen bonds, forming a three-dimensional network stacked down *a*.

## Related literature

Tyrosinase is a key enzyme in the moulting process of insects, see: Kramer & Knost (2001 ▶). For the inhibitory activity on tyrosinase of benzaldehyde thio­semi­carbazones, see: Xue *et al.* (2007 ▶). For the synthesis of the title compound, see: Liu *et al.* (2008 ▶).
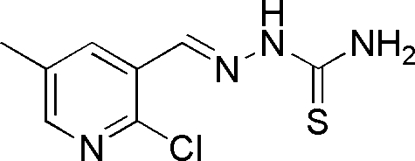


## Experimental

### 

#### Crystal data

C_8_H_9_ClN_4_S
*M* *_r_* = 228.70
Monoclinic, 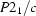

*a* = 8.776 (3) Å
*b* = 15.523 (4) Å
*c* = 7.540 (2) Å
β = 96.193 (16)°
*V* = 1021.2 (5) Å^3^
*Z* = 4
Cu *K*α radiation
μ = 4.95 mm^−1^
*T* = 173 K
0.45 × 0.30 × 0.30 mm

#### Data collection

Rigaku R-AXIS Rapid diffractometer
Absorption correction: numerical (*ABSCOR*; Higashi, 1995 ▶) *T*_min_ = 0.214, *T*_max_ = 0.319
6565 measured reflections
1847 independent reflections
1598 reflections with *I* > 2σ(*I*)
*R*_int_ = 0.048

#### Refinement

*R*[*F* ^2^ > 2σ(*F* ^2^)] = 0.037
*wR*(*F* ^2^) = 0.105
*S* = 1.11
1847 reflections
129 parameters
H-atom parameters constrained
Δρ_max_ = 0.27 e Å^−3^
Δρ_min_ = −0.22 e Å^−3^

### 

Data collection: *RAPID-AUTO* (Rigaku, 2001 ▶); cell refinement: *RAPID-AUTO*; data reduction: *RAPID-AUTO*; program(s) used to solve structure: *SHELXS97* (Sheldrick, 2008 ▶); program(s) used to refine structure: *SHELXL97* (Sheldrick, 2008 ▶); molecular graphics: *XP* in *SHELXTL* (Sheldrick, 2008 ▶); software used to prepare material for publication: *SHELXL97*.

## Supplementary Material

Crystal structure: contains datablocks I, New_Global_Publ_Block. DOI: 10.1107/S1600536810004915/ds2018sup1.cif

Structure factors: contains datablocks I. DOI: 10.1107/S1600536810004915/ds2018Isup2.hkl

Additional supplementary materials:  crystallographic information; 3D view; checkCIF report

## Figures and Tables

**Table 1:** Hydrogen-bond geometry (Å, °)

*D*—H⋯*A*	*D*—H	H⋯*A*	*D*⋯*A*	*D*—H⋯*A*
N3—H3*B*⋯S1^i^	0.88	2.52	3.379 (2)	166
N4—H4*B*⋯N1^ii^	0.88	2.15	3.012 (3)	168
N4—H4*B*⋯Cl1^ii^	0.88	2.98	3.609 (2)	130

Symmetry codes: (i) 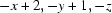; (ii) 
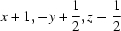
.

**Fig. 1.**
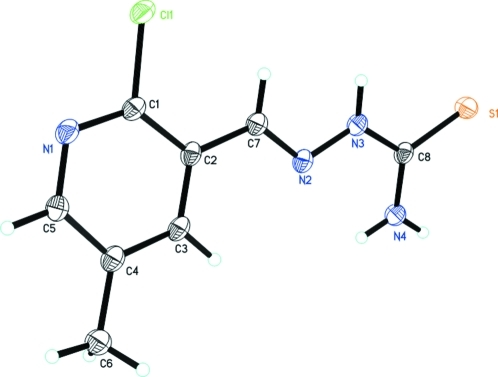
The molecular structure of the title compound , showing the labelling scheme. Displacement ellipsoids are drawn at the 30% probability level for all non-H atoms.

**Fig. 2.**
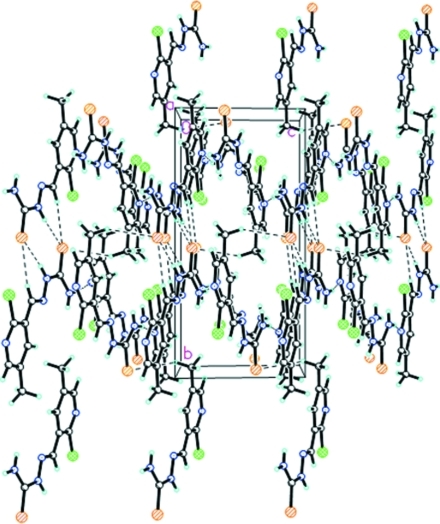
Packing diagram for the title compound viewed along the a axis.

## supplementary crystallographic information

### Comment

Tyrosinase is a key enzyme in the molting process of insect (Kramer & Knost 2001), and benzaldehyde thiosemicarbazones have inhibitory activity on tyrosinase (Xue *et al.*, 2007). In order to look for highly potent tyrosinase inhibitors, the title compound was synthesized by the reaction of thiosemicarbazide and 2-chloro-5-methylnicotinaldehyde (Liu *et al.*, 2008). Finally in the preliminary bioassay, we found that it showed obvious inhibitory activity against tyrosinase from cotton bollworm. To get more information about the structure, we prepared a single crystal of the title compound and its crystal will be reported herein.

The bond distances between N2 and C7 is 1.277 (3) Å, which is in the range of typical bond length of imine double bond. The bond distance of 1.683 (2) Å for the thiocarbonyl group (S1–C8) is about the average value of the typical C=S double bond (1.56 Å) and C–S single bond (1.82 Å), showing a partial double bond character in feature. The partial double bond character also appears between N3 and C8 as well as N4 and C8, which show the distance of 1.355 (3) and 1.322 (3) Å, respectively. In the cryatal structure, there are three intermolecular hydrogen bonds: N3–H3···S1, N4–H4···N1, N4–H4···Cl1 (Table 1).

### Experimental

1.6 g (10 mmol) 2-Chloro-5-methylnicotinaldehyde was dissolved in anhydrous ethanol (15 ml). To this solution, 0.91 g (10 mmol) thiosemicarbazide and 0.5 mL acetic acid were added. The mixture was refluxed for 24 h and then cooled to room temperatur. The precipitate was formed and collected after filteration. The title compound was obtained in 89% yield after recrystallization of the precipitate from anhydrous MeOH. The colourless crystals suitable for X-ray crystallography was carefully grown from anhydrous methanolic solution.

### Refinement

All H atoms were placed in geometrically idealized positions(C—H = 0.93-0.96 Å, N—H=0.86 Å) and treated as riding on their parent atoms, with U_iso_(H) = 1.2-1.5U_eq_(C,*N*).

### Crystal data

**Table:**

C_8_H_9_ClN_4_S	*F*(000) = 472
*M**_r_* = 228.70	*D*_x_ = 1.488 Mg m^−^^3^
Monoclinic, *P*2_1_/*c*	Cu *K*α radiation, λ = 1.54186 Å
Hall symbol: -P 2ybc	Cell parameters from 658 reflections
*a* = 8.776 (3) Å	θ = 3.1–66.2°
*b* = 15.523 (4) Å	µ = 4.95 mm^−^^1^
*c* = 7.540 (2) Å	*T* = 173 K
β = 96.193 (16)°	Block, colorless
*V* = 1021.2 (5) Å^3^	0.45 × 0.30 × 0.30 mm
*Z* = 4	

### Data collection

**Table:**

Rigaku R-AXIS Rapid diffractometer	1847 independent reflections
Radiation source: rotating anode	1598 reflections with *I* > 2σ(*I*)
graphite	*R*_int_ = 0.048
ω scans at fixed χ = 45°	θ_max_ = 68.3°, θ_min_ = 5.1°
Absorption correction: numerical (*ABSCOR*; Higashi, 1995)	*h* = −10→9
*T*_min_ = 0.214, *T*_max_ = 0.319	*k* = −18→17
6565 measured reflections	*l* = −8→9

### Refinement

**Table:**

Refinement on *F*^2^	Secondary atom site location: difference Fourier map
Least-squares matrix: full	Hydrogen site location: inferred from neighbouring sites
*R*[*F*^2^ > 2σ(*F*^2^)] = 0.037	H-atom parameters constrained
*wR*(*F*^2^) = 0.105	*w* = 1/[σ^2^(*F*_o_^2^) + (0.0431*P*)^2^ + 0.4188*P*] where *P* = (*F*_o_^2^ + 2*F*_c_^2^)/3
*S* = 1.11	(Δ/σ)_max_ = 0.001
1847 reflections	Δρ_max_ = 0.27 e Å^−^^3^
129 parameters	Δρ_min_ = −0.22 e Å^−^^3^
0 restraints	Extinction correction: *SHELXL*, Fc^*^=kFc[1+0.001xFc^2^λ^3^/sin(2θ)]^-1/4^
Primary atom site location: structure-invariant direct methods	Extinction coefficient: 0.0051 (7)

### Special details

**Table:**

Geometry. All esds (except the esd in the dihedral angle between two l.s. planes) are estimated using the full covariance matrix. The cell esds are taken into account individually in the estimation of esds in distances, angles and torsion angles; correlations between esds in cell parameters are only used when they are defined by crystal symmetry. An approximate (isotropic) treatment of cell esds is used for estimating esds involving l.s. planes.
Refinement. Refinement of *F*^2^ against ALL reflections. The weighted *R*-factor wR and goodness of fit *S* are based on *F*^2^, conventional *R*-factors *R* are based on *F*, with *F* set to zero for negative *F*^2^. The threshold expression of *F*^2^ > σ(*F*^2^) is used only for calculating *R*-factors(gt) etc. and is not relevant to the choice of reflections for refinement. *R*-factors based on *F*^2^ are statistically about twice as large as those based on *F*, and *R*- factors based on ALL data will be even larger.

### Fractional atomic coordinates and isotropic or equivalent isotropic displacement parameters (Å^2^)

**Table:**

	*x*	*y*	*z*	*U*_iso_*/*U*_eq_	
Cl1	0.50443 (6)	0.31852 (4)	0.16529 (8)	0.0385 (2)	
S1	1.21803 (7)	0.48033 (4)	−0.12061 (9)	0.0413 (2)	
N1	0.5116 (2)	0.15225 (14)	0.1900 (3)	0.0341 (5)	
N2	0.9510 (2)	0.28984 (13)	−0.0128 (2)	0.0309 (5)	
N3	1.0139 (2)	0.36918 (13)	−0.0313 (3)	0.0349 (5)	
H3B	0.9687	0.4145	0.0097	0.042*	
N4	1.2069 (2)	0.31191 (13)	−0.1757 (3)	0.0380 (5)	
H4A	1.1645	0.2609	−0.1669	0.046*	
H4B	1.2914	0.3171	−0.2281	0.046*	
C1	0.5935 (3)	0.21864 (16)	0.1461 (3)	0.0308 (5)	
C2	0.7391 (2)	0.21399 (15)	0.0899 (3)	0.0289 (5)	
C3	0.7996 (3)	0.13151 (16)	0.0783 (3)	0.0315 (5)	
H3A	0.8979	0.1246	0.0385	0.038*	
C4	0.7192 (3)	0.05922 (17)	0.1237 (3)	0.0336 (5)	
C5	0.5753 (3)	0.07344 (16)	0.1798 (3)	0.0357 (6)	
H5A	0.5186	0.0249	0.2129	0.043*	
C6	0.7836 (3)	−0.03035 (16)	0.1176 (3)	0.0407 (6)	
H6A	0.8908	−0.0302	0.1699	0.061*	
H6B	0.7779	−0.0498	−0.0067	0.061*	
H6C	0.7241	−0.0694	0.1854	0.061*	
C7	0.8228 (3)	0.29122 (16)	0.0529 (3)	0.0340 (5)	
H7A	0.7804	0.3455	0.0787	0.041*	
C8	1.1438 (3)	0.38067 (15)	−0.1105 (3)	0.0305 (5)	

### Atomic displacement parameters (Å^2^)

**Table:**

	*U*^11^	*U*^22^	*U*^33^	*U*^12^	*U*^13^	*U*^23^
Cl1	0.0293 (3)	0.0389 (4)	0.0493 (4)	0.0043 (2)	0.0141 (3)	0.0027 (2)
S1	0.0337 (4)	0.0357 (4)	0.0589 (4)	−0.0051 (3)	0.0259 (3)	−0.0051 (3)
N1	0.0275 (10)	0.0380 (11)	0.0387 (11)	0.0010 (9)	0.0119 (8)	0.0023 (9)
N2	0.0255 (10)	0.0368 (11)	0.0313 (10)	−0.0025 (8)	0.0063 (8)	0.0003 (8)
N3	0.0256 (10)	0.0369 (12)	0.0449 (12)	−0.0032 (8)	0.0155 (9)	−0.0046 (9)
N4	0.0283 (11)	0.0369 (12)	0.0519 (13)	−0.0026 (8)	0.0184 (9)	−0.0073 (9)
C1	0.0266 (12)	0.0381 (14)	0.0284 (11)	0.0031 (10)	0.0057 (9)	−0.0013 (9)
C2	0.0242 (11)	0.0374 (14)	0.0263 (11)	−0.0030 (9)	0.0075 (9)	−0.0010 (9)
C3	0.0230 (11)	0.0434 (14)	0.0293 (12)	0.0010 (10)	0.0080 (9)	−0.0014 (10)
C4	0.0290 (12)	0.0422 (14)	0.0307 (12)	0.0001 (10)	0.0080 (9)	−0.0007 (10)
C5	0.0311 (13)	0.0357 (14)	0.0422 (13)	−0.0027 (10)	0.0122 (10)	0.0020 (10)
C6	0.0385 (14)	0.0381 (15)	0.0474 (15)	0.0015 (11)	0.0138 (12)	0.0007 (11)
C7	0.0283 (12)	0.0341 (13)	0.0413 (14)	−0.0017 (10)	0.0115 (10)	−0.0002 (10)
C8	0.0230 (11)	0.0371 (14)	0.0325 (12)	−0.0019 (9)	0.0078 (9)	−0.0004 (9)

### Geometric parameters (Å, °)

**Table:**

Cl1—C1	1.749 (2)	C2—C3	1.392 (3)
S1—C8	1.683 (2)	C2—C7	1.449 (3)
N1—C1	1.319 (3)	C3—C4	1.388 (3)
N1—C5	1.350 (3)	C3—H3A	0.9500
N2—C7	1.277 (3)	C4—C5	1.393 (3)
N2—N3	1.363 (3)	C4—C6	1.503 (3)
N3—C8	1.355 (3)	C5—H5A	0.9500
N3—H3B	0.8800	C6—H6A	0.9800
N4—C8	1.322 (3)	C6—H6B	0.9800
N4—H4A	0.8800	C6—H6C	0.9800
N4—H4B	0.8800	C7—H7A	0.9500
C1—C2	1.391 (3)		
			
C1—N1—C5	117.0 (2)	C3—C4—C6	122.5 (2)
C7—N2—N3	114.1 (2)	C5—C4—C6	120.8 (2)
C8—N3—N2	122.2 (2)	N1—C5—C4	123.7 (2)
C8—N3—H3B	118.9	N1—C5—H5A	118.1
N2—N3—H3B	118.9	C4—C5—H5A	118.1
C8—N4—H4A	120.0	C4—C6—H6A	109.5
C8—N4—H4B	120.0	C4—C6—H6B	109.5
H4A—N4—H4B	120.0	H6A—C6—H6B	109.5
N1—C1—C2	125.4 (2)	C4—C6—H6C	109.5
N1—C1—Cl1	114.30 (17)	H6A—C6—H6C	109.5
C2—C1—Cl1	120.30 (19)	H6B—C6—H6C	109.5
C1—C2—C3	115.8 (2)	N2—C7—C2	123.2 (2)
C1—C2—C7	121.2 (2)	N2—C7—H7A	118.4
C3—C2—C7	123.0 (2)	C2—C7—H7A	118.4
C4—C3—C2	121.4 (2)	N4—C8—N3	117.7 (2)
C4—C3—H3A	119.3	N4—C8—S1	123.06 (18)
C2—C3—H3A	119.3	N3—C8—S1	119.29 (18)
C3—C4—C5	116.7 (2)		
			
C7—N2—N3—C8	−175.1 (2)	C2—C3—C4—C6	−178.2 (2)
C5—N1—C1—C2	0.2 (3)	C1—N1—C5—C4	−1.0 (3)
C5—N1—C1—Cl1	−179.23 (17)	C3—C4—C5—N1	0.7 (4)
N1—C1—C2—C3	0.8 (3)	C6—C4—C5—N1	179.4 (2)
Cl1—C1—C2—C3	−179.73 (16)	N3—N2—C7—C2	−177.63 (19)
N1—C1—C2—C7	−177.0 (2)	C1—C2—C7—N2	−174.1 (2)
Cl1—C1—C2—C7	2.5 (3)	C3—C2—C7—N2	8.3 (4)
C1—C2—C3—C4	−1.2 (3)	N2—N3—C8—N4	2.0 (3)
C7—C2—C3—C4	176.6 (2)	N2—N3—C8—S1	−177.57 (16)
C2—C3—C4—C5	0.5 (3)		

### Hydrogen-bond geometry (Å, °)

**Table:**

*D*—H···*A*	*D*—H	H···*A*	*D*···*A*	*D*—H···*A*
N3—H3B···S1^i^	0.88	2.52	3.379 (2)	166
N4—H4B···N1^ii^	0.88	2.15	3.012 (3)	168
N4—H4B···Cl1^ii^	0.88	2.98	3.609 (2)	130

Symmetry codes: (i) −*x*+2, −*y*+1, −*z*; (ii) *x*+1, −*y*+1/2, *z*−1/2.
